# The Multifunctional Roles of Aquaporins in Tumors: Focusing on Metabolism, Migration, and Regulation of the Tumor Microenvironment

**DOI:** 10.3390/ijms27073016

**Published:** 2026-03-26

**Authors:** Kexin Qu, Rui Wang, Yingwei Bi, Yuxin Liu, Bolin Yi, Jianbo Wang

**Affiliations:** Department of Urology, The First Affiliated Hospital of Dalian Medical University, Dalian 116011, China; qkx3141@163.com (K.Q.); leynad1214@hotmail.com (R.W.); biyingwei_@outlook.com (Y.B.); 19845733936@163.com (Y.L.); yibl@dmu.edu.cn (B.Y.)

**Keywords:** aquaporins, metabolic reprogramming, cell migration, tumor microenvironment, molecular targets

## Abstract

Aquaporins (AQPs) are transmembrane channel proteins that transport water and small solutes. Their dysregulation in cancer reveals functions beyond maintaining osmotic balance. This review summarizes that AQPs drive tumor progression through three core mechanisms: metabolic reprogramming, enhanced motility, and remodeling of the immune microenvironment. Specifically, AQP3, AQP7, and AQP9 serve as metabolic hubs for glycerol, while AQP3 and AQP8 help maintain redox homeostasis. AQP1 and AQP4 facilitate cell migration via hydrodynamic mechanisms, and AQP5 promotes invasion through signaling pathways such as Ras/NF-κB. In immune regulation, AQP9 and AQP3 modulate immune cell function by transporting metabolites, and AQP1 influences angiogenesis. Other isoforms, including AQP0, AQP2, AQP6, AQP10, and AQP11, also play roles in malignancy. Collectively, AQPs form a multifunctional network linking tumor metabolism, physical properties, and immunity, offering insights for novel diagnostic and therapeutic strategies. However, tissue-specific functions, complex regulatory mechanisms, and challenges in developing targeted therapies remain significant hurdles in translational medicine.

## 1. Introduction

Aquaporins (AQPs) are a family of channel-forming membrane proteins. They regulate the passage of water and small solutes and are involved in maintaining osmotic balance and fluid homeostasis [1]. In addition, some AQPs can transport other substances, such as glycerol and H_2_O_2_. Through this, they may play roles in metabolism, signaling, and environmental adaptation [2,3].

Recently, several studies have reported that AQP expression is aberrant in various kinds of cancers. These include glioma [4,5], breast cancer [6,7], lung cancer [8,9], liver cancer [10,11], gastric cancer [12], renal cancer [13], and pancreatic cancer [14]. This dysregulation is linked to tumorigenesis, progression, metastasis, and prognosis. Therefore, AQPs have attracted increasing attention in cancer research [15,16,17]. The roles of AQPs in tumor biology are multifaceted. For instance, AQPs can act as metabolic hubs, promote cell motility, and modify the immune microenvironment. Specifically, AQP3, AQP7, and AQP9 serve as metabolic hubs for glycerol, while AQP3 and AQP8 help maintain redox homeostasis. AQP1 and AQP4 facilitate cell migration via hydrodynamic mechanisms, and AQP5 promotes invasion through signaling pathways such as Ras/NF-κB. In immune regulation, AQP9 and AQP3 modulate immune cell function by transporting metabolites, and AQP1 influences angiogenesis [18,19,20,21,22,23,24,25,26,27,28,29,30]. Hence, AQPs may link tumor cell biophysical traits, metabolism, and interactions with the immune microenvironment.

Previous reviews often describe each AQP subtype in tumors individually. Although these descriptions are very detailed, they do not emphasize the shared biological functions and collaborative roles of these AQPs in tumor progression. Here, we summarize research on AQPs in tumors according to three major aspects: cell migration and invasion, metabolic reprogramming, and immune microenvironment remodeling. We analyze the roles of AQP3, AQP7, AQP8, and AQP9 in glycerol metabolism and redox balance. We discuss the functions of AQP1, AQP4, and AQP5 in promoting migration and invasion. We also examine the roles of AQP1, AQP3 and AQP9 in modulating the immune microenvironment. In addition, the functions of other AQPs (AQP0, AQP2, AQP6, AQP10 and AQP11) in tumors are briefly addressed. Overall, this review provides insight into the complex roles of AQPs in tumorigenesis. It supports their potential as diagnostic biomarkers and therapeutic targets for cancer.

## 2. Regulation of Tumor Metabolic Reprogramming by Aquaporins

**Figure 1:**
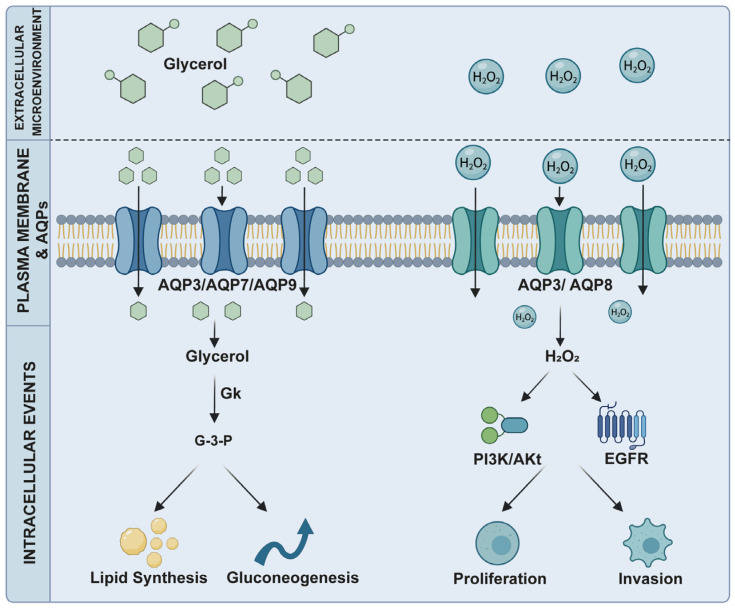
AQPs in tumor metabolic reprogramming.

Metabolic reprogramming is a hallmark of cancer. It provides essential precursors, energy, and redox balance for rapid proliferation and tumor growth [31,32,33]. Some AQPs (AQP3, AQP7, AQP8, AQP9, and AQP10) transport metabolic substances, such as glycerol and H_2_O_2_. This function links them to tumor metabolism and progression [34,35,36,37,38]. This section discusses the role of glycerol metabolism and redox regulation mediated by AQP3, AQP7, AQP8, and AQP9 in tumor cells (Figure 1).

### 2.1. Glycerol Metabolic Hubs: AQP3, AQP7, and AQP9

Lipid metabolic reprogramming is fundamental for tumor growth and determines therapy sensitivity [39]. Glycerol is a substrate for gluconeogenesis and lipogenesis. Its membrane permeability modulates metabolic flux [40]. Once glycerol enters the cell, it is first converted to glycerol-3-phosphate (G3P) by glycerol kinase (GK) [20]. G3P can either enter glycolysis or gluconeogenesis to generate ATP for energy or serve as a key precursor for the synthesis of phospholipids and triglycerides, providing structural materials for the rapid proliferation of tumor cells [20,41]. AQP3, AQP7 and AQP9 are the main aquaglyceroporins responsible for glycerol trafficking. They show specific expression patterns in organs and tumors [34,42,43,44].

AQP3 is overexpressed in numerous cancers and directly promotes lipid synthesis and energy metabolism in tumor cells [18,45]. For example, in gastric cancer, AQP3 promotes lipid synthesis and autophagy, which inhibits apoptosis and drives tumor growth and metastasis [18]. Additionally, studies have found that AQP3-deficient mice exhibit resistance to tumor growth and reduced epidermal proliferation in a skin carcinogenesis model, with significantly decreased levels of glycerol metabolism and ATP content in the skin. Oral glycerol administration reverses these effects, suggesting that AQP3-facilitated glycerol transport promotes tumor progression by enhancing ATP generation [45]. In colon cancer cells, the hormone analog dDAVP specifically downregulates AQP3-mediated glycerol transport via the V1a receptor (V1aR), thereby inhibiting cell growth [46]. Notably, V1aR expression is significantly reduced in colon adenocarcinoma tissues, suggesting that this regulatory axis is disrupted during tumorigenesis [46]. These findings position the vasopressin-AQP3 pathway as a novel targetable node in cancer.

AQP7 expression is mainly observed in adipocytes. It is associated with glycerol release during lipolysis and is involved in systemic lipid metabolism [47]. Its expression is closely associated with obesity and metabolic syndrome. In obese mouse models, its downregulation promotes adipose tissue remodeling and inflammation [48]. A study on breast cancer first identified AQP7 as a key metabolic hub through unbiased network analysis, functioning as a glycerol channel to drive metabolic reprogramming. Subsequent functional experiments and animal models confirmed that AQP7 plays a critical role in breast cancer growth and metastasis, suggesting its involvement in tumor progression through metabolic reprogramming [19]. However, in hepatocellular carcinoma, increased AQP7 expression decreases lipid accumulation, alleviates drug resistance, and improves the immune microenvironment. In contrast, reduced AQP7 expression is related to sorafenib resistance. This suggests heterogeneity among different tumor types in which AQP7 plays a role [49]. A key gap in our knowledge is that we do not yet know which upstream signals or downstream factors determine whether AQP7 acts to “promote” or “suppress” cancer in a specific tumor. Understanding this mechanism is essential for precisely targeting it as a treatment.

AQP9 is mainly expressed in the liver. It transports glycerol in hepatocytes, regulating systemic energy homeostasis [43,50]. For example, one study demonstrated that AQP9 expression shows a close positive correlation with [^14^C]glycerol uptake through in vitro [^14^C]glycerol uptake assays and mouse tumor models, suggesting that aquaglyceroporins could serve as a novel target for cancer molecular imaging [51]. In an IP rat model of early hepatocarcinogenesis, overall liver AQP9 expression was up-regulated and accompanied by enhanced glycerol metabolism. In contrast, AQP9 and GK were significantly down-regulated within preneoplastic foci, suggesting that lesion cells limit glycerol uptake and metabolism, possibly avoiding G3P-related toxicity and gaining a survival advantage [20].

### 2.2. Redox Balance: AQP3 and AQP8

Redox imbalance is key for tumor initiation and progression, affecting proliferation, survival, and therapy resistance [52,53]. Reactive oxygen species (ROS), mainly H_2_O_2_, are involved in this process and have a dual effect. Low concentrations act as signal molecules for tumor progression, whereas high levels cause cell damage [21,54]. AQP3 and AQP8, being permeable to H_2_O_2_, are implicated in regulating redox balance in cancer cells.

AQP3, a peroxiporin, mediates H_2_O_2_ transport, enabling redox signaling between intracellular and extracellular compartments [55,56]. In cervical cancer, AQP3 promotes H_2_O_2_ influx, activating the Syk/PI3K/Akt pathway and enhancing invasion and metastasis [21]. In lung adenocarcinoma, AQP3 increases ROS levels, inactivating PTEN and activating the AKT/mTOR pathway. This inhibits autophagy and promotes cell proliferation [57]. In breast cancer, H_2_O_2_ transported by AQP3 regulates cell migration via the CXCL12/CXCR4-dependent signaling pathway [58]. Studies have shown that AQP3-mediated H_2_O_2_ can also serve as a second messenger to stimulate downstream proliferative pathways of EGFR, promoting cancer cell growth and migration in A431 and H1666 cell lines [59]. Additionally, In the context of gastric carcinogenesis, *Helicobacter pylori* infection upregulates AQP3 expression via activation of the ROS–HIF-1α axis, and the AQP3-mediated H_2_O_2_ uptake in turn amplifies ROS signaling, thereby forming a positive feedback loop (ROS–HIF-1α–AQP3–ROS), suggesting a potential novel mechanism for inflammation-mediated carcinogenesis [60].

AQP8 is located on both the plasma membrane and the mitochondrial membrane. It is directly involved in redox regulation in the mitochondrial matrix [61]. Upregulation of AQP8 increases ROS levels in glioma, activates the AKT pathway, and promotes proliferation, migration, and invasion [62]. In addition, AQP8-mediated H_2_O_2_ transport plays an important role in leukemia cell proliferation. In B1647 acute myeloid leukemia cells, on the one hand, AQP8 mediates the transmembrane influx of H_2_O_2_ generated by NAD(P)H oxidase in response to VEGF stimulation. This regulates the sulfenation level of PTEN, induces PTEN inactivation, and activates the downstream Akt signaling pathway. On the other hand, AQP8 also mediates the transmembrane transport of Nox-derived H_2_O_2_, leading to increased intracellular ROS levels, which in turn activates the phosphorylation of PI3K and p38 MAPK signaling pathways. Together, these signaling events promote the proliferation of leukemia cells [22,23].

AQP3 has been functionally characterized as a peroxiporin in cervical, lung, and breast cancers. Evidence from gene silencing and overexpression studies supports its role in H_2_O_2_-mediated PI3K/AKT activation during tumor progression [21,57,58]. AQP8, in contrast, remains relatively understudied, with its role explored only in glioma and leukemia [22,23,62]. As previously described, the transmembrane transport of H_2_O_2_ mediated by AQP3 and AQP8 plays a critical role in activating oncogenic signaling pathways such as Akt and PI3K, as well as in maintaining the redox homeostasis of tumor cells. Therefore, inhibiting the function or expression of AQP3 and AQP8 may theoretically block their pro-tumor effects, making them potential anticancer targets. However, for this target, Pellavio et al. identified a regulatory mechanism in HeLa cells that differs from the action mode of traditional inhibitors: cerium oxide nanoparticles (CNPs) significantly enhanced the permeability of AQP3 and AQP8 (especially AQP8) to H_2_O_2_. Immunofluorescence colocalization analysis revealed a clear interaction between CNPs and AQP3 as well as AQP8; functional experiments further confirmed that CNPs treatment increased the AQP-mediated transmembrane transport rate of H_2_O_2_, and this effect was abolished after AQP8 silencing, suggesting that AQP8 is a key molecule in the regulatory effect of CNPs [56]. Of note, tumor cells rely on moderate levels of ROS to sustain survival, and overcorrection of the redox status may disrupt their signaling homeostasis, thereby inhibiting proliferation and inducing cell death [63]. For instance, a study on melanoma showed that CNPs mediated tumor cell death by increasing H_2_O_2_ levels [64]. However, no study to date has directly established a causal link between the enhancing effect of CNPs on AQP channel permeability and their anti-tumor activity, and the underlying mechanisms require further functional validation. In summary, we speculate that AQP3 and AQP8, as H_2_O_2_ channels, possess functional duality in tumor biology—their ultimate effect depends on the direction and flux of H_2_O_2_ transport, as well as the redox context of the cells. By enhancing rather than inhibiting channel permeability, CNPs may offer a strategy for targeting AQPs in anti-tumor therapy that differs from traditional inhibitors. However, the specific mechanisms and therapeutic value remain to be further explored.

### 2.3. Summary

In conclusion, AQPs contribute to tumor progression through the transport of two key substrates: glycerol and H_2_O_2_. Glycerol influx fuels energy production and lipid synthesis, meeting the high biosynthetic demands of proliferating cancer cells. Meanwhile, H_2_O_2_ transport serves as a second messenger, activating downstream oncogenic signaling cascades—including PI3K/Akt, MAPK, and EGFR pathways—thereby promoting proliferation, migration, and survival. Together, these metabolic and signaling functions reshape the tumor microenvironment. Targeting these metabolism-related AQPs thus represents a promising therapeutic strategy.

## 3. AQP-Mediated Tumor Cell Migration and Invasion

**Figure 2:**
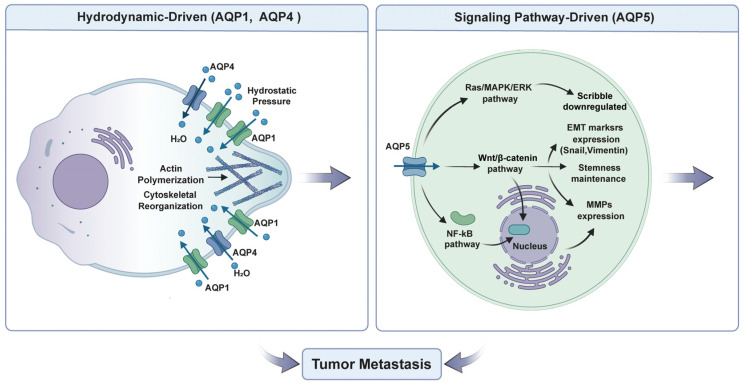
AQPs in tumor cell migration and invasion.

Tumor cell migration and invasion are two crucial stages during tumor infiltration and metastasis. These processes include cytoskeleton remodeling, alteration of cell–matrix adhesion, and activation of signaling pathways [65,66,67]. Recent research has confirmed that AQPs participate in the above processes via their transmembrane transport function [15,24,68]. Based on different mechanisms, AQPs can be classified as “hydrodynamic-driven” (AQP1, AQP4) [24,25,26,69] or “signaling pathway-driven” (AQP5) [70]. (Figure 2).

### 3.1. Hydrodynamic-Driven: AQP1 and AQP4

Water transport through AQP1 and AQP4 regulates tumor cell migration. They do so by controlling water fluxes and regulating membrane protrusions, lamellipodia extensions, and cell volume changes, which provide the physical driving force for movement.

Overexpression of AQP1 occurs in gliomas, gastric cancer, triple-negative breast cancer, and lung cancer. It is associated with increased tumor invasiveness and poor prognosis [71,72,73,74,75]. The pro-migratory mechanism of AQP1 combines physical and molecular signaling. First, AQP1’s core function is to rapidly regulate transmembrane water transport, providing the fundamental physical driving force for cell migration. Previous studies have demonstrated that osmotic and hydrostatic pressures, arising from water flow, drive changes in cell shape and motility [76]. By enhancing membrane water permeability, AQP1 facilitates water influx and efflux along osmotic gradients. In cirrhotic angiogenesis, AQP1 increases local water permeability in membrane blebs, driving bleb expansion and retraction to promote amoeboid invasion of endothelial cells [69]. During zebrafish vascular development, AQP1a.1 (the homolog of AQP1) enriches at the leading edge of migrating tip cells. Water influx generates hydrostatic pressure that favors actin polymerization, accelerating filopodia extension and stabilizing protrusions to synergistically drive tip cell migration [77]. In neuroblastoma, hypoxia upregulates AQP1 and polarizes it to leading-edge lamellipodia. Enhanced local water permeability drives rapid water influx, causing anterior swelling and posterior shrinkage to promote directional migration [24,25].

AQP1 also activates important signaling pathways such as the Wnt/β-catenin pathway, which can promote migration and invasion. In breast cancer, AQP1 upregulation promotes activation of the Wnt/β-catenin pathway. This leads to increased expression of Epithelial–mesenchymal transition (EMT) markers and acquisition of cancer stemness properties [78]. In rheumatoid arthritis, inhibition of the Wnt/β-catenin pathway by XAV939 abrogates the oncogenic effect caused by AQP1. Conversely, activation of the Wnt/β-catenin pathway with lithium chloride rescues cell migratory ability. This further confirms the central role of the AQP1→Wnt/β-catenin axis in driving cell migration and invasion [79]. More importantly, AQP1 interacts with ANXA2 and Rab1b in breast cancer, leading to the release of metastasis-promoting proteins and increased invasiveness [80].

AQP4 is predominantly found in the central nervous system, specifically in the end-feet region of astrocytes surrounding blood vessels [81]. It is associated with peritumoral edema and tumor invasion behavior [82,83,84]. The role of AQP4 is to control cell volume dynamics to drive tumor cell migration. In highly invasive glioma cells, AQP4 expression is significantly upregulated. This upregulation is directly correlated with accelerated transmembrane water exchange, biophysically linking AQP4’s water channel function to a migratory phenotype [26]. Evidence from an astrocyte differentiation model (neural stem cells) shows that upregulation of AQP4 expression during differentiation increases water transport rate, while AQP4 knockout significantly slows cell migration. This indicates that AQP4 drives cell migration by enhancing cell membrane water permeability [85]. Importantly, AQP4’s function extends beyond water permeability. Its supramolecular aggregation state critically regulates migration patterns: the tetrameric form promotes more effective collective astrocyte migration than larger orthogonal arrays of particles (OAPs), indicating that AQP4 organization influences cytoskeletal coordination and mechanosensing to determine migration efficiency and mode [86].

AQP4 expression can be regulated by multiple pathways in the tumor microenvironment. Bradykinin activates the MEK1-ERK1/2-NF-κB pathway, upregulates AQP4 expression, and promotes glioma migration [87]. LINC00461 can upregulate AQP4 expression to promote glioma cell proliferation and chemoresistance [88]. AQP4 can also cooperates with MMPs to promote tumor migration and invasion in glioma [89]. In addition, circ_0079530 regulates AQP4 through the miR-409-3p axis and affects tumor progression in non-small cell lung cancer [90].

### 3.2. Signaling Pathway-Driven: AQP5

AQP5 is upregulated in various cancers such as lung, colorectal, prostate, gastric, and ovarian cancer. It is consistently associated with poor prognosis [27,91,92,93,94]. In tumors, its primary function is to act as a key signaling hub that activates multiple downstream oncogenic pathways, directly or indirectly, thereby driving tumor cells to acquire an invasive phenotype [70,95,96]. The oncogenic mechanism of AQP5 involves the integration of multiple pathways, as outlined at the three levels below.

First, AQP5 can activate major oncogenic signaling pathways, including Ras/MAPK, NF-κB, and Wnt/β-catenin. For instance, in breast cancer, AQP5 overexpression activates Ras signaling, increases cell motility, and detaches cells from the main tumor mass [70]. In non-small cell lung cancer, AQP5 maintains phosphorylation of the EGFR/ERK1/2 pathway, promoting cell motility and angiogenesis [27]. In hepatocellular carcinoma, AQP5 activates NF-κB to control EMT and metastasis [97]. In colorectal cancer, AQP5 activates NF-κB, enhancing cell proliferation and chemoresistance [98]. In triple-negative breast cancer, AQP5 activates Wnt/β-catenin, upregulates EMT markers and accelerates cell migration and invasion [96].

Second, AQP5 plays a role in maintaining tumor stem cell characteristics and controlling autophagy. In gastric cancer, AQP5 is abundant in stem cells. It promotes autophagy and self-renewal via TRPV4 and Wnt/β-catenin/NF-κB signaling [99,100]. AQP5 interacts with ISL1 to induce FOXO signaling. It facilitates nuclear localization of FOXO3 and upregulation of stemness-related genes such as CD44 [101]. In gastric cardia adenocarcinoma, NNMT-mediated metabolic reprogramming activates the Wnt/β-catenin pathway, maintains the stemness of AQP5+ cells, and drives tumorigenesis [100].

Third, AQP5 disrupts cell polarity and mediates microenvironment communication. In breast cancer, AQP5 downregulates the polarity protein Scribble, disorganizes epithelial polarity, and promotes migration [70]. Tumor cells transport AQP5 into endothelial cells through extracellular vesicles, which stimulates angiogenesis and facilitates metastasis [102]. In gastric cancer, AQP5+ tumor stem cells interact with DHX9, deplete arginine, and impair NK cell activity, enabling immune escape [103].

### 3.3. Summary

The role of AQPs in tumor migration and invasion is subtype-dependent, reflecting distinct mechanistic functions. AQP1 and AQP4 act as “hydrodynamic engines,” driving migration by facilitating rapid water transport and cell volume regulation to generate the biophysical force for membrane protrusions and directional movement. This function is supported by multiple in vivo and in vitro studies, including genetic models (e.g., zebrafish AQP1a.1) and cellular assays [26,69,77,82,83,84,86]. In contrast, AQP5 serves as a “signaling commander” that promotes invasion by activating key oncogenic pathways such as Ras/MAPK, NF-κB, and Wnt/β-catenin, as demonstrated in overexpression and silencing experiments across various cancer types [27,70,96,97,98].

In conclusion, AQP1 and AQP4 act as “hydrodynamic engines” that control water flow and cell volume regulation, laying the physiological foundation for migration. In contrast, AQP5 acts as a “signaling commander” that links important signaling cascades to facilitate invasion, stemness, EMT, and microenvironment reprogramming. Although these three types of AQPs have distinct functions, they together form a complex regulatory network that sustains tumor metastasis. Further understanding of this network would be beneficial for designing anti-metastasis drugs that block particular AQPs or their specific functions.

## 4. AQP-Mediated Remodeling of the Tumor Immune Microenvironment (TIME)

**Figure 3:**
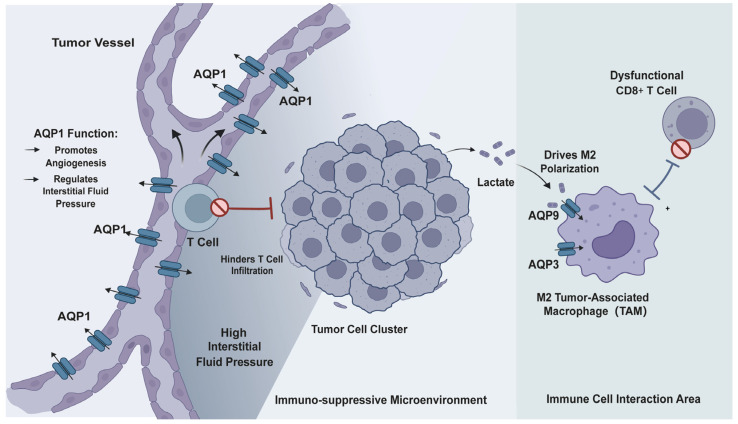
AQPs in remodeling the tumor immune microenvironment.

The tumor immune microenvironment primarily refers to the network of immune cells, immunomodulatory molecules, and their interactions with tumor cells. It coexists with the broader tumor microenvironment (TME), which includes blood vessels, fibroblasts, and extracellular matrix, collectively influencing tumor progression. All of these significantly influence tumor progression, metastasis, and therapy response [104,105]. Aquaporins not only affect tumor cell behavior but also play a crucial role in reshaping the immunosuppressive TIME. They do so by modulating immune cell function and local angiogenesis [28,29,30,106,107]. This section discusses the roles of AQP1, AQP3, and AQP9 in this process (Figure 3).

### 4.1. Immune Cell Metabolism and Function Regulation: AQP9 and AQP3

The functional state of immune cells is closely linked to their metabolic profile [108,109]. AQP9 and AQP3 are involved in tumor metabolism and immune responses by transporting metabolites such as lactate, glycerol, and H_2_O_2_, thereby influencing immune cell function and polarization.

AQP9 is expressed in various immune cells, including macrophages and neutrophils, and functions as an important immune regulator [110,111]. In colon cancer, tumor-associated macrophages (TAMs) take up lactate from glycolytic tumor cells via AQP9. This lactate uptake drives metabolic reprogramming and M2 polarization in macrophages, promoting tumor growth, angiogenesis, and immune escape. Macrophage-specific deletion of AQP9 prevents these effects and inhibits tumor progression [107]. Under hypoxic conditions, AQP9 is transcriptionally activated by HIF-1α, further enhancing M2 macrophage polarization, suppressing CD8+ T cell cytotoxicity, and accelerating colon cancer progression [28]. In breast cancer, tumor-associated neutrophils promote tumor cell proliferation and migration/invasion by upregulating AQP9, which activates STAT3 phosphorylation to establish the AQP9/STAT3 signaling axis [112]. Bioinformatics analyses have shown that AQP9 expression correlates positively with infiltration of CD8+ T cells, macrophages, and dendritic cells in multiple cancers, including clear cell renal cell carcinoma (ccRCC), head and neck squamous cell carcinoma, and lung cancer. High AQP9 expression is often associated with an immune-enriched microenvironment and poor prognosis [110,113,114,115]. Interestingly, in laryngeal cancer, AQP9 acts as a tumor suppressor, inhibiting proliferation, migration, and invasion, and correlating with favorable outcomes [116]. This dual role may stem from differences in AQP9 expression in different cell types (such as immune cells versus tumor cells) and the complexity of its substrates (including lactate, glycerol, and possibly even drugs). Future research should use advanced techniques like single-cell spatial transcriptomics to analyze the specific expression and function of AQP9 in different components of the tumor ecosystem. This will help explain these contradictions and support the development of cell-type-specific therapies.

AQP3 also contributes to the regulation of the tumor immune microenvironment [117]. In lung adenocarcinoma, scRNA-seq shows AQP3 is enriched in M2 macrophages and promotes their polarization via the PPAR-γ/NF-κB axis; these AQP3-high M2 macrophages upregulate IL-6 to drive tumor progression, growth, and migration [29]. In a preclinical colon cancer model, anti-AQP3 monoclonal antibodies reduced M2 macrophage polarization, reactivated T cell proliferation, and exerted anti-tumor effects [118]. Beyond regulating macrophages, AQP3 shapes the tumor immune landscape and influences therapeutic responses. For instance, in chronic myeloid leukemia, AQP3 is one of four diagnostic genes; a risk score based on it can distinguish molecular subtypes and predict response to imatinib [119].

### 4.2. Angiogenesis and Interstitial Fluid Pressure Regulation: AQP1

AQP1 not only promotes tumor cell migration but also plays a key role in angiogenesis and regulation of interstitial fluid pressure (IFP), both of which indirectly influence tumor invasion and metastasis.

Abnormal vascular structure and elevated IFP are hallmarks of the TIME that limit immune cell infiltration and drug delivery [120]. AQP1 plays a dual central role in this context. First, AQP1 directly promotes tumor angiogenesis. In gastric cancer, AQP1 expression positively correlates with VEGF, and tissues positive for either show higher microvessel density [72]. Functionally, AQP1 is a key downstream effector for TRPC5-mediated angiogenesis; its silencing abolishes the pro-angiogenic effect of TRPC5 [121]. In diabetic nephropathy models, hirudin reduces the expression of AQP1 and VEGFA/VEGFR2, thereby suppressing pathological angiogenesis [122]. AQP1 itself can modulate endothelial angiogenesis via H_2_O_2_ transport affecting the CaMKII-AMPK pathway [123] and interacts with the ERK MAPK pathway under mifepristone [124]. Furthermore, in glioblastoma, AQP1 is involved in tumor malignancy by promoting the formation of vascular beds that are characteristic of glioblastoma by downregulating THSD7A [30].

In addition, AQP1 regulates water transport across the vascular wall, thereby modulating IFP and consequently contributing to the formation of an immunosuppressive microenvironment. AQP1 is overexpressed on both apical and basolateral membranes of endothelial cells in fenestrated capillaries, enabling bidirectional water flux and maintaining interstitial fluid balance [125]. A study on atherosclerosis found that blocking or knocking down AQP1 expression significantly reduces the total hydraulic conductivity of the vascular wall, meaning the rate of water transport across the vessel decreases [126]. And Wu et al. using mathematical modeling and parameter sensitivity analysis, concluded that changes in vascular hydraulic conductivity are a direct cause of alterations in tumor IFP [127]. Therefore, we speculate that AQP1 influences tumor IFP by modulating the hydraulic conductivity of the vascular wall. Elevated IFP is a hallmark of solid tumors; it compresses blood vessels, impedes penetration of large therapeutic molecules, and inhibits immune cell infiltration [128,129,130]. High IFP within a certain range (such as 40 mmHg) has also been shown to regulate the YAP/BMF signaling axis, thereby maintaining the stemness of liver cancer stem cells [131]. Thus, AQP1 promotes tumor progression through two interrelated mechanisms: enhancing angiogenesis and sustaining high IFP, which together create a microenvironment conducive to tumor growth and immune evasion.

### 4.3. Summary

The role of AQP9 in the tumor immune microenvironment is emerging as a research focus [107,110,111]. Shi et al. provided strong functional evidence using macrophage-specific AQP9 knockout mice, demonstrating its role in promoting M2 polarization and tumor progression through lactate transport [107]. AQP1’s involvement in angiogenesis is supported by multiple disease models, including glioblastoma and diabetic nephropathy [30,122]. It is worth noting that there is currently no direct evidence showing that AQP1 regulates IFP. However, through the mathematical model and parameter sensitivity analysis established by Wu et al., we found that vascular hydraulic conductivity is a determining factor for tumor IFP, and AQP1 is a key molecule regulating this conductivity. Therefore, we speculate that AQP1 may influence IFP through this pathway [126,127]. This speculation remains to be validated by experiments involving direct intervention of AQP1 and measurement of IFP changes.

In conclusion, AQPs regulate the TIME through two mechanisms: immune metabolism (AQP9 and AQP3) and physical barriers (AQP1). By modulating lactate and H_2_O_2_ transport, AQP9 and AQP3 influence macrophage function and polarization. AQP1 promotes angiogenesis and regulates IFP, thereby limiting immune cell infiltration and drug delivery. Together, these pathways construct a tumor microenvironment that supports tumor growth and immune escape, offering potential targets for therapies aimed at reversing immunosuppression and improving drug delivery.

## 5. Potential Functions and Mechanisms of Other AQP Members in Tumors

In addition to the major AQP subtypes discussed above (AQP1, AQP3, AQP4, AQP5, AQP7, AQP8, and AQP9), other members of the AQP family—AQP2, AQP6, AQP10 and AQP11—may also play roles in cancer. Although research on these AQPs is limited, emerging evidence suggests their involvement in tumor progression through metabolic, migratory, or microenvironmental interactions. This section briefly reviews these AQPs and their potential contributions to tumor biology.

### 5.1. AQP0

AQP0 mRNA expression has been detected in the retina, liver, and testicular Sertoli cells, and shows high expression in lens fiber cells [132]. Shen et al. found via RT-PCR that AQP0 mRNA was absent in both human gastric carcinoma tissues and the corresponding normal tissues [12]. Furthermore, research by Thapa et al. indicates that AQP0 mRNA expression is associated with poorer survival in all gastric cancer patients [93]. This association is particularly evident in patients with intestinal-type cancer (both male and female) and in those with clinical stage I and III disease.

### 5.2. AQP2

AQP2 is primarily expressed in the kidney collecting duct, where it regulates water reabsorption in response to antidiuretic hormone [133]. Recent studies have reported abnormal AQP2 expression in urinary system tumors. Low AQP2 expression in ccRCC correlates with advanced tumor stage and poor prognosis, suggesting a tumor suppressor role [134,135]. Mechanistically, AQP2 binds to a micropeptide encoded by lncRNA MIAC, inhibiting EGFR signaling and suppressing PI3K/AKT and MAPK activity, thereby reducing tumor cell proliferation and migration [136]. In prostate cancer, AQP2 is an exosome-associated prognostic gene, and its expression pattern correlates with suppressed tumor progression and treatment response [137]. Thus, AQP2 may represent a potential therapeutic target in cancer. Notably, elevated AQP2 expression in pheochromocytoma and paraganglioma is associated with increased tumor size, underscoring its functional heterogeneity [138].

### 5.3. AQP6

AQP6 is an aquaporin that exhibits both anion and H_2_O_2_ channel activity [139,140]. In malignant pleural mesothelioma (MPM), AQP6 mediates stress-induced H_2_O_2_ release, enabling tumor cells to evade oxidative stress and ferroptosis, thereby contributing to chemoresistance [140]. Furthermore, studies indicate that CNPs can influence H_2_O_2_ permeability in HeLa cells by modulating the function of AQP3, AQP6 and AQP8, suggesting that targeting AQP6 could influence oxidative stress conditions in tumors [56].

### 5.4. AQP10

AQP10 is mainly expressed in the small intestine, where it participates in water and glycerol absorption [141]. Research on AQP10 in cancer is limited. Bioinformatics analyses indicate that AQP10 may be associated with tumor prognosis and immune regulation. In ccRCC, low expression of AQP10 correlates with shorter overall survival and immune cell infiltration, suggesting a role in immune modulation [142]. AQP10 transcription is also downregulated in advanced gastric cancer [143]. These findings suggest that reduced AQP10 expression may indirectly influence the immune microenvironment, although the underlying mechanisms require experimental validation.

### 5.5. AQP11

AQP11 is a non-classical water channel localized to the endoplasmic reticulum, where it is involved in H_2_O_2_ transport and maintenance of redox homeostasis [144]. In colorectal cancer, AQP11 expression is downregulated, and its overexpression inhibits CRC cell proliferation, migration, and invasion, while silencing AQP11 promotes these phenotypes. AQP11 expression is negatively regulated by miR-152-3p [145]. Furthermore, studies in diabetic nephropathy models have shown that zinc oxide nanoparticles can simultaneously upregulate AQP11 expression, inhibit the mTOR signaling pathway, and activate autophagy [146]. We speculate that this metabolic regulation pattern may also exist in tumor cells, suggesting a potential role for AQP11 in tumor metabolic reprogramming.

### 5.6. Summary

Although AQP0, AQP2, AQP6, AQP10 and AQP11 have not been extensively studied in cancer, existing evidence indicates that they participate in tumor-promoting processes beyond traditional growth-related mechanisms. For example, AQP2 and AQP11 may influence signaling pathways, while AQP6 and AQP10 may regulate the tumor microenvironment. Further investigation into these “non-essential” AQP family members may reveal their roles in tumor migration, invasion, metabolic reprogramming, and microenvironment remodeling and whether they can serve as organ-specific diagnostic biomarkers or therapeutic targets to complement current AQP-based therapies.

## 6. Conclusions and Perspectives

**Figure 4:**
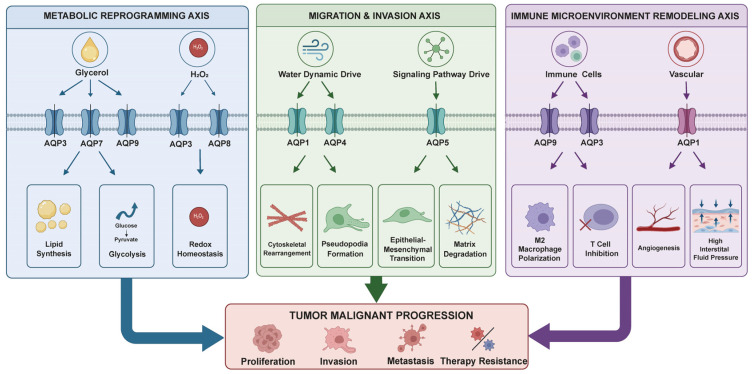
A multifunctional network of AQPs in tumor progression.

In summary, current evidence strongly indicates that AQPs are more than just simple water channels. They have emerged as versatile and multi-faceted key regulators in tumor biology. By integrating metabolic reprogramming, physical migration, and remodeling of the immune microenvironment, they form a complex network that promotes tumor progression (Figure 4, Table 1). We posit that the roles of AQPs in tumors are not isolated; rather, they constitute a critical interface connecting the intrinsic properties of tumor cells with the external microenvironment.

However, our understanding of this network remains in its early stages. The apparent contradictions in existing evidence—such as the dual roles of AQP7 and AQP9—precisely reveal the highly context-dependent and complex nature of their functions, which is a direct reflection of tumor heterogeneity. Therefore, they should no longer be simply labeled as “oncogenes” or “tumor suppressors.” Instead, they should be viewed as functional executors whose roles depend on specific spatiotemporal and microenvironmental contexts. Future research must fully embrace this complexity. More advanced techniques are required to analyze the dynamic changes within the AQP network at single-cell and spatial resolution.

Currently, research on AQP inhibitors is at the preclinical stage. The main strategies include gene silencing, biologics, small molecule modulation, and nanoparticle modulation, with specific progress summarized below (Table 2).

(1) Gene Silencing Technologies: The use of siRNA or shRNA targeting AQP1, AQP3, and AQP5 has been validated in in vitro and in vivo studies to inhibit tumor growth and metastasis [18,73,91,96]. (2) Biologics: A monoclonal antibody targeting AQP3 has shown anti-tumor effects in a colon cancer model by modulating macrophage polarization [118]. (3) Small Molecule Modulation: The hormone analog dDAVP can downregulate AQP3-mediated glycerol transport via the V1a receptor, thereby inhibiting tumor cell growth [46]. (4) Nanoparticle Modulation: Cerium oxide nanoparticles can modulate AQP8-mediated H_2_O_2_ permeability, affecting the oxidative stress status of tumor cells [56].

Despite certain progress, the clinical translation of AQP-targeted therapies still faces challenges. (1) Off-Target Effects: Some AQPs have important physiological functions in normal tissues, and targeted therapy may cause off-target toxicity. (2) Targeted Delivery Challenges: How to specifically deliver drugs to tumor-associated cell populations (such as tumor-associated macrophages) remains an urgent issue to be addressed. (3) Context-Dependent Functions: Some AQPs may play opposite roles in different tumor types. For example, AQP7 promotes tumor growth in breast cancer [19], but may alleviate drug resistance and improve the immune microenvironment in hepatocellular carcinoma [49]. This context-dependent functionality suggests that therapeutic strategies targeting AQPs must carefully consider tumor type, cell subpopulation, and microenvironmental context, as indiscriminate targeting may yield unintended consequences.

Future research directions may include: (1) Functional Mechanism Dissection: In-depth investigation of how non-coding RNAs, post-translational modifications, and interacting proteins regulate AQP activity, stability, and subcellular localization. (2) Exploration of Combination Therapies: Evaluating the effects of AQP-targeted therapies combined with chemotherapy, radiotherapy, or immunotherapy in preclinical models. (3) Development of Biomarkers: Identifying AQP expression signatures or circulating AQP-positive extracellular vesicles for patient selection and treatment response prediction.

In conclusion, AQPs, as multifunctional molecules connecting tumor metabolism, migration, and immune regulation, have transformed from passive water channels to promising therapeutic targets. However, the field still needs to move from descriptive studies toward mechanistic investigations and rigorous translational research. Overcoming the challenges of tumor heterogeneity, functional complexity, and targeted delivery will be key to achieving the clinical translation of AQP-targeted therapies.

## Figures and Tables

**Table 1:** Multifunctional Roles of AQPs in Tumors.

AQP Isoform	Primary Functions in Tumors	Key Mechanisms	Associated Cancers	Clinical Relevance	Refs.
AQP0	May correlates with patient prognosis	Unknown	Gastric cancer (particularly the intestinal type)	Poor prognostic marker	[12,93]
AQP1	Promotes cell migration, angiogenesis, and may regulates IFP	1. Facilitates water flux for membrane protrusion and motility; 2. Activates Wnt/β-catenin signaling to induce EMT; 3. Enhances endothelial cell migration and sprouting; 4. Modulates vascular wall water permeability and may affect IFP	Glioma, gastric cancer, breast cancer, lung cancer	Prognostic marker; potential target for anti-angiogenic and anti-metastatic therapy	[24,69,71,72,73,74,75,78,121,126,127]
AQP2	May act as a tumor suppressor	Binds to micropeptide MIAC, inhibiting EGFR signaling and downstream PI3K/AKT/MAPK pathways	ccRCC, prostate cancer	Prognostic marker	[134,136,137]
AQP3	Regulates metabolism (glycerol, H_2_O_2_), immune modulation, and promotes migration/invasion	1. Mediates glycerol uptake for lipid synthesis and ATP production; 2. Transports H_2_O_2_, activating PI3K/AKT and other redox-sensitive pathways; 3. Promotes M2 macrophage polarization via PPAR-γ/NF-κB	Breast cancer, gastric cancer, cervical cancer, colorectal cancer, lung adenocarcinoma	Key metabolic and immune modulator; potential target for monoclonal antibody therapy	[18,21,45,57,118]
AQP4	Drives cell migration	Controls water transport for cell volume dynamics and invasion	Glioma	Contributor to glioma invasion	[26,82,84,89]
AQP5	Acts as a signaling hub, promotes stemness, EMT, migration, and invasion	1. Activates oncogenic pathways (Ras/MAPK, NF-κB, Wnt/β-catenin). 2. Maintains cancer stem cell properties via Wnt/β-catenin/NF-κB signaling. 3. Disrupts cell polarity	Lung cancer, colorectal cancer, gastric cancer, breast cancer, hepatocellular carcinoma	Strong prognostic marker for poor outcome; potential target to inhibit metastasis and chemoresistance	[27,70,91,92,93,94,96,97,99,100]
AQP6	Confers resistance to oxidative stress and chemotherapy	Mediates H_2_O_2_ efflux, protecting cells from ferroptosis and oxidative damage	Malignant pleural mesothelioma	Potential contributor to chemotherapy resistance	[140]
AQP7	Regulates glycerol metabolism and lipid metabolic reprogramming	Facilitates glycerol influx, involved in lipid metabolism and energy homeostasis	Breast cancer, hepatocellular carcinoma.	Dual role (pro-tumor in breast cancer; may alleviate drug resistance in HCC)	[19,47,48,49]
AQP8	Modulates redox balance, promotes proliferation	1. Channels H_2_O_2_ across mitochondrial and plasma membranes; 2. Increases ROS levels, activating AKT pathway	Glioma, leukemia	Potential target to enhance sensitivity to radiotherapy or oxidative stress-inducing therapies	[22,23,62]
AQP9	Mediates glycerol and lactate transport, reprograms immune metabolism	1. Transports lactate into TAMs, driving M2 polarization and immunosuppression; 2. Facilitates glycerol uptake for gluconeogenesis/lipogenesis in hepatocytes; 3. Activates STAT3 signaling in tumor-associated neutrophils to promote tumor progression; 4. Expression regulated by HIF-1α under hypoxic conditions	Colon cancer, hepatocellular carcinoma, breast cancer, ccRCC, glioma	Dual role (pro-tumor or suppressor) depending on context; key immune-metabolic interface and prognostic indicator	[28,44,51,107,110,112,113]
AQP10	Potential role in immune regulation	Bioinformatics associations with immune cell infiltration; exact mechanism unclear	ccRCC, gastric cancer	Candidate immune microenvironment biomarker; requires functional validation	[142,143]
AQP11	May suppress tumor growth	Overexpression inhibits proliferation and migration	Colorectal cancer	Potential tumor suppressor	[145]

**Table 2:** Potential Therapeutic Strategies Targeting AQPs in Cancer.

AQP Target	Therapeutic Strategy	Key Mechanism/Agent	Refs.
AQP1	Gene silencing	siRNA/shRNA knockdown	[73]
AQP3	Monoclonal antibody	Anti-AQP3 mAb blocks immune modulation	[118]
	Gene silencing	siRNA knockdown	[18]
	Small molecule modulation	dDAVP downregulates AQP3-mediated glycerol transport via V1aR	[46]
AQP5	Gene silencing	siRNA/shRNA knockdown of oncogenic hub	[91,96]
AQP8	Nanoparticle modulation	CNPs modulate H_2_O_2_ flux (requires validation)	[56]
AQP9	Genetic targeting	Macrophage-specific AQP9 knockout	[107]

## Data Availability

No new data were created or analyzed in this study. Data sharing is not applicable to this article.

## Abbreviations

The following abbreviations are used in this manuscript:

AQP	Aquaporin
BBB	Blood–brain barrier
ccRCC	Clear cell renal cell carcinoma
CNS	Central nervous system
CRC	Colorectal cancer
EMT	Epithelial–mesenchymal transition
HCC	Hepatocellular carcinoma
IFP	Interstitial fluid pressure
MPM	Malignant pleural mesothelioma
PDAC	Pancreatic ductal adenocarcinoma
ROS	Reactive oxygen species
TAMs	Tumor-associated macrophages
V1aR	V1a receptor
CNPs	Cerium oxide nanoparticles
GK	Glycerol kinase
G3P	glycerol-3-phosphate
TIME	Tumor immune microenvironment
